# Decoding Waterpipe Tobacco Smoking: A Comprehensive Narrative Review Exploring Mechanics, Health Risks, Regulatory Challenges, and Public Health Imperatives

**DOI:** 10.7759/cureus.52168

**Published:** 2024-01-12

**Authors:** Sankalp Yadav

**Affiliations:** 1 Medicine, Shri Madan Lal Khurana Chest Clinic, New Delhi, IND

**Keywords:** hookah smoking, passive smoking, smoking tobacco, shisha, waterpipe tobacco smoking

## Abstract

Waterpipe tobacco smoking, commonly known as hookah or shisha, has witnessed a surge in popularity globally, particularly among young adults. However, this activity is associated with several issues related to health. This comprehensive narrative review aims to provide a valuable resource for researchers, policymakers, and public health professionals seeking to deepen their understanding of waterpipe tobacco smoking and implement evidence-based strategies to address its challenges.

The present article focuses on an in-depth analysis of the health risks associated with waterpipe tobacco smoking. Based on evidence from the current scientific literature, the review explores the impact of waterpipe smoking on respiratory health, cardiovascular outcomes, and the potential links to infectious diseases. Additionally, the review discusses the emerging evidence on the long-term health consequences, highlighting the need for continued research in this area. Also, it touches on the regulatory challenges surrounding waterpipe tobacco smoking, which were critically examined, emphasizing the gaps and inconsistencies in existing policies. Finally, the review underscores the public health imperatives necessitated by the rise of waterpipe tobacco smoking. The narrative concludes by proposing a holistic approach that integrates scientific evidence, regulatory frameworks, and public health initiatives to mitigate the growing impact of waterpipe tobacco smoking on population health.

## Introduction and background

Tobacco smoking has been a global public health concern for decades, with various forms of smoking contributing to a significant burden of preventable diseases and mortality [1]. Among the diverse methods of tobacco consumption, waterpipe tobacco smoking, commonly known as hookah, hubble-bubble, kalian, nargeela, argeela, goza, borry, qaylan, chica, arghileh, sheesha, okka, nargileh, boury, ghelyoon, ghalyan, gouza, mada’a, or shisha, has emerged as a unique and increasingly popular practice [2,3].

Background of waterpipe tobacco smoking

The roots of waterpipe smoking can be traced back centuries, originating in the Middle East and India [2,4-6]. Initially, the waterpipe was a cultural and social tradition, fostering a sense of community and relaxation. Over time, its practice has transcended geographical boundaries, evolving into a global phenomenon [3]. Traditional waterpipes consist of a bowl to hold tobacco, a water-filled base, a hose, and a mouthpiece [7]. The tobacco is heated using charcoal, and the resulting smoke passes through the water before being inhaled [8].

While waterpipe smoking has a rich cultural history, its contemporary resurgence is marked by a transformation from a regional tradition to a globalized trend. This shift has been fueled by various factors, including globalization, migration, and the influence of popular culture. As a result, waterpipe smoking is no longer confined to specific cultural or geographic contexts but has permeated diverse communities worldwide [3].

Popularity and social perception

In recent years, waterpipe smoking has gained traction, particularly among young adults and university students [9]. The allure of waterpipe establishments lies not only in the act of smoking itself but also in the social and recreational aspects that accompany it. The communal nature of waterpipe sessions fosters a sense of camaraderie, providing a venue for socializing, conversation, and relaxation [10,11].

The perception of waterpipe smoking as a less harmful alternative to conventional cigarette smoking has contributed significantly to its popularity. Many individuals believe that the water filtration process removes harmful substances, making it a seemingly safer option [12]. Additionally, the flavored tobacco used in waterpipes, often marketed as a variety of enticing flavors, adds an element of novelty, attracting individuals who may be dissuaded by the taste of traditional cigarettes [11].

Purpose of the review

Amid the rising popularity of waterpipe tobacco smoking, it is crucial to critically examine the practice and its associated health implications. This review aims to provide a comprehensive analysis of waterpipe smoking, shedding light on its mechanics, health effects, and regulatory challenges. By exploring the nuances of waterpipe smoking, the aim is to inform both the general public and policymakers about the potential risks and misconceptions surrounding this practice.

Through a meticulous examination of scientific literature, epidemiological studies, and regulatory frameworks, this review intends to contribute to a nuanced understanding of waterpipe tobacco smoking. By elucidating the complex interplay between cultural traditions, societal perceptions, and health outcomes, it is aimed at facilitating informed decision-making among individuals, healthcare professionals, and policymakers. Additionally, the review highlights the importance of public health interventions and evidence-based policies to address the challenges posed by the growing prevalence of waterpipe smoking in contemporary society.

For this narrative review, the following electronic databases were searched: Cochrane Library, MEDLINE, EMBASE, PsycINFO, Web of Science, and CINAHL Plus with Full Text. Search terms included "waterpipe" and its many variant terms, "hubble bubble," "shisha," "hookah," "goza," "arghileh," and "narghile." The search was limited to papers published in English, between January 1, 1990, and January 1, 2024. 

## Review

Mechanics of waterpipe smoking

Components of a Waterpipe

Understanding the mechanics of waterpipe smoking involves examining the intricate components that contribute to the inhalation of tobacco smoke. A traditional waterpipe typically comprises a bowl, a water-filled base, a hose, and a mouthpiece [7]. The bowl contains flavored tobacco, known as maassel, which is heated using charcoal. As the tobacco combusts, the resulting smoke passes through the water in the base before being drawn through the hose and inhaled through the mouthpiece [8].

Bowl: The bowl is the receptacle for the flavored tobacco. It is where the tobacco is placed and heated for smoking [8].

Water-filled base: The base of the waterpipe is filled with water, and it serves multiple purposes. Contrary to popular belief, the primary role of the water is not to filter out harmful substances but to cool the smoke. The cooling effect is achieved as the smoke bubbles through the water, potentially making the inhalation process smoother [13].

Hose: The hose is the conduit through which the smoke travels from the water-filled base to the mouthpiece. It can be made of various materials, including leather, synthetic fabrics, plastic, silicone, or rubber [14].

Mouthpiece: The mouthpiece is where the user inhales the smoke. It is typically detachable for hygiene purposes and is passed around during communal smoking sessions [14,15].

Misconceptions Regarding Harm Reduction

One of the prevailing misconceptions surrounding waterpipe smoking is the belief in its inherent harm reduction compared to traditional cigarette smoking [3]. While the water filtration process may remove some water-soluble components and cool the smoke, it does not eliminate the toxicants present in tobacco. The following are key misconceptions.

Filtering out harmful substances: Many individuals assume that the water in the base filters out harmful substances, providing a false sense of security. However, studies have shown that while some particulate matter is removed, the levels of harmful components, such as carbon monoxide and heavy metals, remain significant [15].

Reduced nicotine intake: Contrary to popular belief, waterpipe smoking is not a nicotine-free activity. Users can still become addicted to nicotine, and the communal aspect of waterpipe sessions may contribute to a normalization of tobacco use, fostering addiction [16].

Flavored tobacco and safety: The use of flavored tobacco in waterpipes is often marketed as a safer alternative. However, the appealing flavors can mask the harshness of the smoke, potentially leading users to inhale more deeply and increasing their exposure to harmful substances [17].

Studies on Smoke Composition

Scientific research has played a crucial role in unraveling the composition of waterpipe smoke and providing insights into the potential health risks associated with this form of tobacco consumption. Studies have utilized advanced analytical techniques to assess the chemical makeup of the smoke and compare it to that of traditional cigarettes [16].

Chemical composition: Research indicates that waterpipe smoke contains a complex mixture of toxicants, including carbon monoxide, heavy metals (such as lead and arsenic), polycyclic aromatic hydrocarbons (PAHs), and volatile organic compounds (VOCs). These substances are known contributors to respiratory and cardiovascular diseases, as well as cancer [18].

Comparison with cigarette smoke: Comparative studies have highlighted that, despite differences in delivery mechanisms, the concentrations of harmful constituents in waterpipe smoke can be comparable to or even higher than those in cigarette smoke. This challenges the notion that waterpipe smoking is a less harmful alternative. Some studies have reported that when compared to cigarette smoking, waterpipe tobacco smoking is associated with greater carbon monoxide (CO), similar nicotine, and dramatically more smoke exposure [19].

By critically examining the components of waterpipe smoking and debunking common misconceptions, it becomes evident that the perceived harm reduction associated with this practice is often unfounded. Understanding the mechanics of waterpipe smoking lays the groundwork for a comprehensive assessment of its health implications, which will be further explored in subsequent sections of this review.

Health implications

Waterpipe tobacco smoking, with its social allure and perceived harm reduction, is not without consequences. The health implications of this practice extend beyond the commonly acknowledged risks associated with cigarette smoking [20]. In the past decade, the prevalence of cigarette smoking decreased by 7.21% but the prevalence of waterpipe smoking increased by 7.80%. The highest population-attributable risk was shown for oesophageal (35.0%), lung (30.50%), and gastric (8.20%) cancers. This section delves into the multifaceted health impacts of waterpipe smoking, examining its association with nicotine addiction, respiratory and cardiovascular effects, infectious risks, and cancer.

Nicotine Addiction

Prevalence and patterns: Waterpipe smoking is not immune to the addictive properties of nicotine. Studies have revealed the prevalence of nicotine dependence among waterpipe users, challenging the notion that waterpipe smoking is a less addictive alternative. The communal nature of waterpipe sessions may contribute to sustained use patterns, and emerging evidence suggests that individuals engaging in waterpipe smoking can develop nicotine addiction over time [21].

Social and cultural factors: The prevalence of waterpipe smoking is influenced by social and cultural factors. The communal aspect of waterpipe sessions, often embedded in cultural and social traditions, can create a sense of social cohesion. However, this very communal nature may foster nicotine addiction, as individuals are more likely to engage in the habit in group settings. The normalization of waterpipe smoking within specific social circles contributes to the perpetuation of addictive behaviors [22].

Respiratory and Cardiovascular Effects

Chronic bronchitis and lung function impairment: Contrary to the perception that waterpipe smoking is less harmful to the respiratory system, research suggests a link between waterpipe use and respiratory issues. Chronic bronchitis, characterized by persistent coughing and mucus production, has been observed in waterpipe smokers. Additionally, studies indicate impaired lung function among individuals engaging in waterpipe smoking, highlighting the potential for long-term respiratory consequences [23].

Cardiovascular issues: The cardiovascular effects of waterpipe smoking extend beyond the respiratory system. Exposure to carbon monoxide and other cardiovascular toxicants in waterpipe smoke can contribute to an increased risk of cardiovascular diseases. The inhalation of these harmful substances places strain on the cardiovascular system, potentially leading to conditions such as hypertension and an elevated risk of heart disease [23,24].

Infectious Risks

Transmission of diseases: The communal nature of waterpipe smoking poses a unique set of infectious risks. Sharing the mouthpiece and hoses during group sessions creates an environment conducive to the transmission of infectious diseases. Studies have demonstrated the potential for the spread of respiratory infections, including tuberculosis and viral infections such as herpes, hepatitis, etc., among waterpipe users [25].

Risks associated with communal smoking: Communal waterpipe smoking settings, such as hookah lounges, may inadvertently contribute to the rapid transmission of contagious diseases. The close proximity of individuals, shared waterpipe equipment, and the exchange of saliva during group sessions increase the likelihood of disease transmission. A number of causative organisms of diseases like herpes simplex virus (HSV-1), Epstein-Barr, *Helicobactor pylori*, hepatitis B, and various respiratory infections, including bacterial meningitis, tuberculosis, and periodontal conditions such as oral candida can spread by these communal waterpipe tobacco smoking sessions. This aspect of waterpipe smoking emphasizes the importance of considering infectious risks beyond individual health impacts [15,23]. 

Cancer Risk

Emerging research findings: Emerging research has begun to shed light on the association between waterpipe smoking and an increased risk of certain cancers. While traditional cigarette smoking is well-established as a leading cause of lung cancer, recent studies suggest that waterpipe smoking may also elevate the risk of lung cancer and other malignancies. The prolonged exposure to carcinogenic compounds in waterpipe smoke contributes to this emerging health concern [26].

Carcinogenic compounds in waterpipe smoke: The smoke produced during waterpipe sessions contains a range of carcinogenic compounds, including PAHs and VOCs. These substances have been linked to the development of various cancers, possibly due to genetic material damage, emphasizing the need for a comprehensive understanding of the cancer risks associated with waterpipe smoking [26]. Table 1 details the components of waterpipe smoke and its health impacts.

**Table 1:** Health impact of components of waterpipe tobacco smoke

Smoke toxicants	Health impact
Tar	Carcinogenic. Narrowing bronchioles and negative impact on cilia
Nicotine	Carcinogenic. Impact on the heart, reproductive system, lung, kidney, etc.
Carbon monoxide	Headache, nausea, rapid breathing, weakness, exhaustion, dizziness, and confusion
Polycyclic aromatic hydrocarbons	Lung, skin, and bladder cancers
Benzo(a)pyrene	Carcinogenic. Skin rash or eye irritation with redness and/or a burning sensation
Dibenz(a,h)anthracene	headache, dizziness, nausea, and vomiting may affect the liver and kidneys
Indeno(1,2,3-cd)pyrene	Carcinogenic. Reproductive damage
Aldehydes	
Formaldehyde	Irritation of the eyes, nose, and throat at low levels for short periods. Longer exposure or higher doses can cause coughing or choking. Severe exposure can cause death
Acetaldehyde	Faster heartbeat, a headache, or an upset stomach. Impairs brain activity and memory
Acrolein	Irritating to the skin, eyes, and mucous membranes. Respiratory distress and delayed pulmonary edema
Heavy metals	
Arsenic	Very high chances of lung and bladder cancer
Chromium	Oral and epidermal allergic contact dermatitis, as well as pulmonary sensitization
Lead	Carcinogenic
Beryllium	Carcinogenic
Nickel	Carcinogenic
Cobalt	Carcinogenic
Cadmium	Carcinogenic
Phenols	
Phenol	Pungent smells are associated with intense irritation and corrosion of the eyes, mucosa, and skin. Affects sensory quality
*o*-Cresol	Liver and kidney damage
*m*-Cresol	Effects on the central nervous system
*p*-Cresol	Irritate the nose, throat, and lungs
Catechol	Tumor promoters
Resorcinol	Irritation of the nose and throat
Hydroquinone	Tumor promoters

As this review delves into the health implications of waterpipe tobacco smoking, it becomes evident that the practice extends beyond the social and cultural dimensions that often shape its perception. The addictive nature of nicotine, coupled with the respiratory, cardiovascular, infectious, and potential cancer risks, underscores the need for increased awareness and evidence-based interventions to address the multifaceted health challenges associated with waterpipe smoking.

Regulatory challenges

The regulatory landscape surrounding waterpipe tobacco smoking poses unique challenges, driven by perceptions of reduced harm and cultural perception that sets it apart from traditional cigarette smoking. This section delves into the complex regulatory challenges associated with waterpipe smoking, exploring existing perceptions, regulatory gaps, comparisons with traditional smoking regulations, the imperative for stricter policies, and the potential effectiveness of measures designed to discourage its use.

Perceptions and Regulatory Gaps

The regulation of waterpipe tobacco smoking faces challenges rooted in widespread misconceptions about its perceived harm reduction compared to traditional cigarette smoking. Many individuals, influenced by marketing and cultural norms, view waterpipe smoking as a less harmful or even harmless social activity. This perception has contributed to regulatory gaps, where waterpipe establishments often escape the stringent regulations applied to traditional smoking venues [27].

Perceived harm reduction: The prevailing perception of waterpipe smoking as a safer alternative to traditional cigarettes has led to a regulatory environment that may underestimate its health risks. Public awareness campaigns and educational efforts are crucial to dispel these misconceptions and ensure that regulatory decisions are grounded in an accurate understanding of the potential harms associated with waterpipe tobacco smoking.

Regulatory gaps: Regulatory frameworks often lag behind the evolving landscape of tobacco consumption. Waterpipe establishments may operate in regulatory gray areas, benefiting from the perception of reduced harm and a lack of specific regulations tailored to address the unique characteristics of waterpipe smoking. Addressing these regulatory gaps requires a comprehensive reassessment of existing policies [11].

Comparisons With Traditional Smoking Regulations

Drawing parallels between waterpipe smoking and traditional cigarette smoking is essential for effective regulation. While both practices involve the inhalation of tobacco smoke, the differences in delivery mechanisms, social contexts, and public perception necessitate nuanced regulatory approaches. Understanding these distinctions is crucial for crafting policies that adequately address the health risks associated with waterpipe tobacco smoking [28].

Inherent differences: Waterpipe smoking often occurs in social settings, creating challenges for enforcement compared to the more individualistic nature of cigarette smoking. Moreover, the perception of waterpipe smoking as a cultural or social activity may influence regulatory decisions. Balancing the need for comprehensive regulations with cultural sensitivity is a delicate but necessary task [28].

Public health impact: Comparing the public health impacts of waterpipe and traditional smoking is vital for informing regulatory decisions. While waterpipe smoking may be less prevalent than cigarette smoking, its potential health risks, coupled with the infectious risks associated with communal sessions, necessitate a careful examination of its societal impact [27,28].

Need for Stricter Policies

The emerging evidence of the health implications associated with waterpipe smoking underscores the urgency for stricter regulatory policies. Existing regulations may not adequately address the unique challenges posed by waterpipe establishments, communal smoking practices, and the misperception of harm reduction. Stricter policies should encompass various aspects, including marketing and advertising restrictions, age verification measures, and smoke-free regulations [29].

Marketing and advertising restrictions: Regulations should curtail the marketing and advertising practices that contribute to the perception of waterpipe smoking as a harmless or trendy activity. Clear guidelines on the promotion of flavored tobacco and the depiction of waterpipe smoking in media can mitigate the appeal of these products, especially among youth [28,29].

Age verification measures: Strengthening age verification measures is paramount to preventing the initiation of waterpipe smoking among minors. Stricter enforcement of age restrictions on the sale and entry into waterpipe establishments can serve as a deterrent and limit access to vulnerable populations [28].

Smoke-free regulations: Extending smoke-free regulations to include waterpipe establishments is essential for safeguarding public health. The risks of secondhand smoke exposure associated with waterpipe smoking necessitate comprehensive smoke-free policies that align with those applied to traditional smoking venues [29].

Implementing Measures to Discourage Use

Discouraging waterpipe tobacco smoking requires a multifaceted approach that extends beyond regulatory frameworks. Implementing measures to discourage use involves leveraging educational campaigns, community engagement, and collaborations with healthcare professionals to convey accurate information about the health risks associated with waterpipe smoking [28,29].

Educational campaigns: Public awareness campaigns should dispel misconceptions about waterpipe smoking, emphasizing its potential health risks and the lack of significant harm reduction compared to traditional cigarettes. Educational initiatives targeting both the general public and healthcare professionals can play a pivotal role in altering perceptions and fostering informed decision-making [30].

Community engagement: Engaging communities, particularly those where waterpipe smoking is deeply entrenched in cultural or social practices, is crucial for effective implementation. Collaborations with community leaders, influencers, and educators can facilitate a nuanced understanding of waterpipe smoking, enabling tailored interventions that respect cultural sensitivities while prioritizing public health [30].

Healthcare professional involvement: Involving healthcare professionals in anti-waterpipe smoking initiatives is essential for reaching individuals at risk and providing evidence-based information. Clinicians can play a vital role in counseling patients on the health risks of waterpipe smoking, offering cessation support, and contributing to broader public health efforts. However, there is a paucity of literature on the perceptions of clinicians on waterpipe smoking cessation [31].

As regulatory challenges persist in the realm of waterpipe tobacco smoking, an integrated approach that combines comprehensive policies with educational initiatives and community engagement is imperative. Striking a balance between cultural sensitivity and evidence-based public health measures will be instrumental in effectively addressing the health risks associated with waterpipe smoking.

Public awareness and education

Public awareness and education play pivotal roles in addressing the challenges posed by waterpipe tobacco smoking. Dispelling misconceptions, engaging healthcare professionals, launching targeted public health campaigns, and implementing education programs in schools and communities are integral components of a comprehensive strategy to curb the prevalence and associated health risks of waterpipe smoking.

Dispelling Misconceptions

Providing accurate information: Central to any public awareness campaign is the provision of accurate information. Dispelling misconceptions requires clear communication about the health risks associated with waterpipe smoking. Educational materials should highlight that the water filtration process does not eliminate harmful substances, and the practice is not a risk-free alternative to traditional cigarette smoking [32].

Addressing perceived harm reduction: Targeting the perception of harm reduction is crucial. Public awareness initiatives should elucidate the similarities between waterpipe smoking and traditional cigarette smoking, emphasizing that both practices pose significant health risks. Comparisons of the smoke composition and associated health outcomes can effectively challenge the prevailing notion that waterpipe smoking is less harmful [33].

Healthcare Professional's Role

Incorporating waterpipe counseling into clinical practice: Healthcare professionals play a vital role in disseminating accurate information about waterpipe smoking to patients. Integrating waterpipe counseling into routine clinical practice enables professionals to discuss the health risks, address misconceptions, and provide support for individuals attempting to quit. This proactive approach fosters a patient-centered dialogue about tobacco use [31,34].

Offering cessation support: Healthcare professionals should be equipped to offer cessation support tailored to waterpipe smokers. This includes evidence-based interventions, counseling services, and resources to aid in quitting. Incorporating waterpipe cessation discussions into routine healthcare appointments can be instrumental in promoting behavioral change, although data is limited [34].

Public Health Campaigns

Targeted messaging: Public health campaigns must employ targeted messaging to reach specific demographics, particularly the youth, and communities where waterpipe smoking is prevalent. The messaging should emphasize the health risks associated with waterpipe smoking, dispel misconceptions, and underscore the potential for addiction. Utilizing culturally sensitive approaches ensures relevance and resonance with diverse populations [33,35].

Media engagement: Leveraging various media channels, including social media, traditional advertising, and community-based outlets, enhances the reach of public health campaigns. Engaging influencers and community leaders can amplify the impact of messages, fostering a community-driven response to waterpipe smoking. Collaborations with media outlets can further contribute to the dissemination of evidence-based information [33].

School and Community Education Programs

Incorporating waterpipe education into school curricula: Education programs in schools should integrate information about waterpipe smoking into existing curricula. Providing students with accurate and age-appropriate information creates awareness early on, dispels misconceptions, and equips them with the knowledge to make informed decisions about tobacco [36].

Community workshops and outreach: Community education programs should extend beyond formal education settings. Hosting workshops, seminars, and community outreach events raises awareness about the health risks of waterpipe smoking. Collaborating with local organizations, community leaders, and healthcare professionals ensures that education programs are culturally sensitive and resonate with diverse populations [37,38].

Peer-to-peer initiatives: Empowering peer-led initiatives within schools and communities fosters a sense of ownership and peer-to-peer influence. Peer educators can play a crucial role in disseminating information, initiating conversations, and promoting behavioral change among their peers, creating a ripple effect in challenging societal norms surrounding waterpipe smoking [39].

In short, public awareness and education initiatives form the cornerstone of a comprehensive strategy to address the challenges associated with waterpipe tobacco smoking. By dispelling misconceptions, engaging healthcare professionals, launching targeted public health campaigns, and implementing education programs in schools and communities, societies can empower individuals with the knowledge needed to make informed decisions about their health.

## Conclusions

In conclusion, the global challenge of waterpipe tobacco smoking necessitates a comprehensive and united approach. To effectively combat this issue, it is imperative to dispel misconceptions through targeted awareness campaigns, fortify regulatory frameworks to curtail accessibility, actively involve healthcare professionals in prevention and cessation efforts, and implement vigorous public education initiatives. By synergizing these multifaceted strategies, societies can make significant strides in reducing the prevalence of waterpipe smoking and alleviating its correlated health risks. The urgency of the matter underscores the need for decisive action, aiming not only to safeguard public health but also to foster informed decision-making among individuals on a global scale.
